# Brain Tumor Imaging with Iopamidol CEST MRI: In Vivo Detection and Validation

**DOI:** 10.1021/cbmi.5c00101

**Published:** 2025-09-23

**Authors:** Elena Botto, Antonella Carella, Francesco Gammaraccio, Daisy Villano, Riccardo Gambino, Alessia Corrado, Elisa Pirotta, Feriel Romdhane, Dario Livio Longo

**Affiliations:** † Institute of Biostructures and Bioimaging (IBB), 9327 National Research Council of Italy (CNR), Via Nizza 52, Torino 10126, Italy; ‡ Department of Molecular Biotechnology and Health Sciences, University of Turin, Via Nizza 52, Turin 10126, Italy

**Keywords:** magnetic resonance imaging, chemical exchange saturation transfer, glioblastoma, iopamidol, gadoteridol

## Abstract

Several diamagnetic CEST (chemical exchange saturation transfer) molecules have been proposed as a potential alternative to gadolinium-based contrast agents (CAs), with promising contrast efficiency properties. However, a direct comparison of CEST contrast agents in brain tumors, where gadolinium CAs are considered the gold standard for detecting primary masses, is still lacking. The aim of this study is to investigate the capability of Iopamidol, a CT contrast medium, to detect and delineate brain tumors in mice using the MRI-CEST technique compared with a conventional gadolinium-based contrast agent. Iopamidol provided enough contrast enhancement to detect and delineate brain tumors in both postinjection and contrast-enhanced images, comparable to Gadoteridol, in a glioblastoma murine model obtained upon stereotaxic injection of GL261 cells into C57BL/6 mice. Quantitative comparison between tumor and healthy tissue was assessed with contrast-to-noise ratio (CNR) and lesion-to-brain ratio (LBR) metrics. LBR values were 2.7-fold larger for Iopamidol than for Gadoteridol, although the CNR values were lower. The diagnostic accuracy of segmented tumor regions on both Iopamidol- and Gadoteridol-derived contrast images was calculated by the Tanimoto, DICE similarity, and volume similarity coefficients that indicated strong similarities between the contoured regions from Iopamidol and Gadoteridol contrast images. Moreover, moderate to excellent agreements were observed for intra- and interobserver variability. Overall, Iopamidol showed a capability similar to that of Gadoteridol to detect and contour the tumor area, with good diagnostic performance in terms of tumor border delineation in brain tumors.

## Graphical abstract

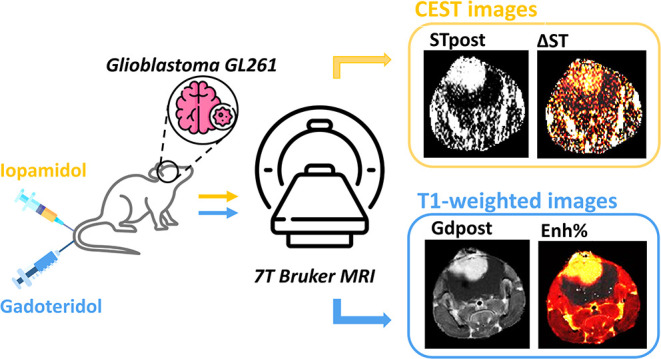

## Introduction

Gliomas are the most common malignant brain lesions, including glioblastoma multiforme, with a less than two-year survival after diagnosis and upon treatment procedures.^1^

Structural MRI plays a crucial role in assessing brain tumors by determining their location, tissue involvement, and impact on surrounding structures.^2^ The administration of Gadolinium-based contrast agents (GBCAs) for contrast-enhanced (CE) MRI has become a common practice to improve the detection, visualization, and border delineation of tumor in the central nervous system.^3,4^

GBCAs are widely used; however, repeated administration leads to an accumulation in the deep nuclei of the brain (globus pallidus and dentate nucleus),^5–9^ limiting the use of certain linear GBCAs in MRI, while macrocyclic agents can still be used at lower doses.^10^

In view of these considerations, alternative contrast agents with higher relaxivities or that do not contain gadolinium have been investigated.^11,12^ High relaxivity agents, like Gadopiclenol and Gadoquatrane, have demonstrated similar diagnostic effects to conventional GBCAs, but at a lower clinical dose.^13–18^ Manganese-based contrast agents, such as Mn-PyC3A, and other metal-based agents have also been evaluated, demonstrating comparable tumor contrast enhancement to GBCAs in preclinical models.^19–25^ Additional strategies to minimize gadolinium-related toxicities have also been explored, by delivering GBCAs throughout peptide- or polymer-based supramolecular structures (including hydrogels and nanogels).^12,26–29^

Diamagnetic molecules have also been investigated within the chemical exchange saturation transfer (CEST) technique as potential MRI-based contrast agents, owing to the contrast induced throughout the selective saturation of mobile proton pools in exchange with the bulk water molecules.^30–32^ Small molecules such as sugars and iodinated contrast media or macromolecules such as dextran or other polymers showed good contrast enhancement properties and potential clinical translation because of their good biocompatibility.^33–40^ Of note, iodinated contrast media have been extensively studied as potential MRI-CEST alternative to gadolinium agents owing to their similar physiochemical properties and pharmacokinetics to GBCAs^41^ and to their high safety profile.^42^

In particular, Iopamidol is administered to millions of patients every year for a variety of clinical applications, including angiography, parenchymal and perfusion imaging,^43^ with a very low rate of acute and severe adverse reaction effects.^44^ The presence within the chemical structure of two not chemically equivalent amide groups with mobile protons in exchange with water allows the selective saturation of these protons that, following the chemical exchange with water proton, reduce the bulk water signal, hence producing the CEST contrast.^45^ Iopamidol has been shown to provide a marked CEST contrast and good pH sensitivity (because of the pH dependence of the chemical exchange mechanism) for pH mapping in several tissues and diseases.^45–53^ Recently, novel approaches for increasing CEST contrast efficiency have been proposed by the incorporation of iodinated contrast media into supramolecular systems.^54–56^

Previous studies have shown comparable tumor detection between iodinated contrast media and small molecular weight GBCA,^57,58^ as well as between macromolecular CEST and gadolinium-based contrast agents,^34,38,59^ but they were limited to subcutaneous tumor models and by assessing only contrast enhancement properties, thus providing modest information regarding the clinical translatability of the observed findings for brain tumor detection.

The current preclinical study used a murine glioblastoma model to evaluate the contrast efficacy of Iopamidol for detecting primary brain lesions and for delineating tumor borders and compared it with that of a commercially available GBCA.

## Experimental Section

### Animal Model

Animal manipulation and experimental procedures were carried out in accordance with the European Community guidelines (directive 2010/63) and under the approval of the ethics committee of the Ministry of Health (authorization #532/2019).

The GL261 murine glioblastoma cells were cultured in DMEM (Dulbecco’s modified Eagle’s medium) supplemented with 1% l-glutamine, 10% fetal bovine serum, and 1% penicillin/streptomycin. For the orthotopic intracerebral implantation, animals were anesthetized by injecting of a mixture of xilatina 5 mg/kg (Rompum, Bayer) and tiletamine/zolazepam 20 mg/kg (Zoletil 100, Virbac), and fixed in a stereotactic frame and 2.5 × 10^4^ GL261 cells (volume = 10 μL) were slowly injected with a Hamilton syringe into the left hemisphere (1.5 mm ML from the bregma and 3.0 mm DV from the dura) in 8 weeks old male C57BL/6 mice (*n* = 10 Charles River Laboratories, Calco, Italy). Animals were imaged 30 days after tumor induction. All the animals received sequential administration of Iopamidol (Isovue, Bracco Imaging; dose: 4 g iodine/kg body weight, corresponding to 10 mmol Iopamidol/kg bw.) and of Gadoteridol (ProHance, Bracco Imaging) injection (dose: 0.2 mmol Gd/kg b.w.) with a 30 min time interval.^60^ Both contrast agents (Figure 1) were administered intravenously through the tail vein with a catheter with a 29-gauge needle by using an MRI-compatible PHD 22/2000 syringe pump (Harvard Apparatus) during the MRI protocol.

**Figure 1:**
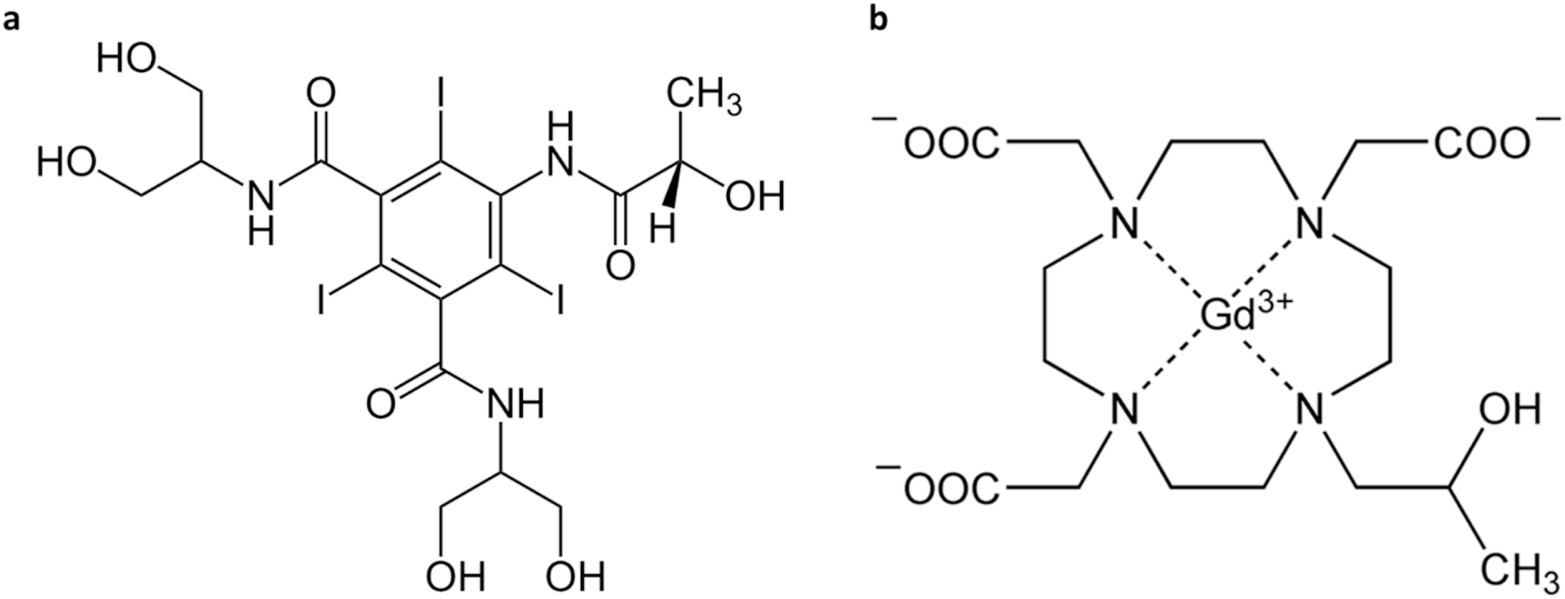
Chemical structure of Iopamidol (a) and Gadoteridol (b).

### MRI Protocol

Mice were anaesthetized with the same intramuscular solution as described above. The MRI study was performed with a 7T microimaging Avance NEO scanner (Bruker) equipped with a dedicated 1H quadrature mouse brain coil. The MRI protocol (Figure 2) started with a localizer scan followed by a series of axial, coronal, and sagittal T2-weighted images for setting the high-resolution anatomical volume in the axial direction (Fast Spin Echo, repetition time of 4000 ms, echo time of 5.9 ms, average: 2, field of view of 20 × 20 mm^2^, matrix: 256 × 256, 8 slices at 1.5 mm slice thickness, scan time of 2.13 min).

**Figure 2:**
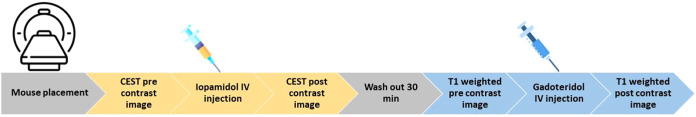
Flowchart of the MRI acquisition protocol.

CEST images were acquired before and after Iopamidol injection with a multislice fast spin echo sequence with centric encoding (repetition time of 12000 ms, echo time of 3.77 ms, field of view of 20 × 20 mm^2^, matrix: 96 × 96 reconstructed to 128 × 128, 8 slices at 1.5 mm slice thickness, 1 average), upon irradiation of 46 frequencies unevenly spaced in the range 10 ppm from the bulk water signal (saturation power: 3 T, saturation duration: 3s + 1s) for an overall acquisition time of 10 min.^61^

T1-weighted images were acquired before and 5 min after Gadoteridol injection with a gradient echo sequence (axial 2D fast low angle shot, FLASH sequence, repetition time of 78 ms, echo time of 1.8 ms, flip angle 45°, field of view of 20 × 20 mm^2^, matrix: 128 × 128, 8 slices at 1.5 mm slice thickness, 10 averages) with the same orientation and spatial resolution of the CEST images, for an overall acquisition time of 2 min.

### Image Analysis

All MRI images were analyzed using in-house scripts written in MATLAB (R2022b; Mathworks, Inc.). CEST images were analyzed pixel-by-pixel by interpolating the Z-spectra by smoothing splines, B0 shift corrected, and by asymmetry analysis for calculating the CEST contrast (saturation transfer—ST%) at 4.2 ppm before (STpre) and after (STpost) Iopamidol injection. Subtraction of the CEST contrast between post- and pre-Iopamidol injection allowed to calculate the difference contrast map (ΔST) for removing the endogenous contributions.^62^ T1-weighted images were analyzed by calculating the percentage increase in signal intensity enhancement (Enh%) between pre- (Gdpre) and post- (Gdpost) Gadoteridol injection as follows:

$$Enh\;\%=\frac{{SI}_{post}-{SI}_{pre}}{{SI}_{pre}}\times 100$$

where SI_post_ indicates signal intensity (SI) on postinjection T1-weighted images and SI_pre_ indicates the signal intensity on preinjection T1-weighted images.

### Qualitative Evaluation

All images were qualitatively evaluated by two readers (D.L.L. and E.B., with at least five years of experience in preclinical MRI imaging) blinded to the type of the injected contrast agent. Visual degree of contrast enhancement and tumor border delineation were evaluated in CEST postinjection contrast images (STpost) and in T1-weighted postinjection images (Gdpost) with a score from 1 to 4 (from “no or unclear delineation/no enhancement” to “clear border delineation/brightly enhanced”).^14^ The mean scores given by the two readers were reported.

### Quantitative Evaluation

Region of interest (ROIs) were manually placed in area of contrast enhancement within the tumor and in an adjacent healthy brain tissue (Supporting Information Figure S1) in both CEST images (STpost) and in T1-weighted images (Gdpost).

The following quantitative metrics for comparing the diagnostic efficacy were calculated based on signal intensity for T1-weighted images after Gadoteridol injection (SIpost) and on ST contrast after Iopamidol injection (STpost) for CEST images:

The contrast-to-noise ratio (CNR) between tumor and healthy brain tissue:

$$\mathrm{for}\;T1-\mathrm{weighted}\;\mathrm{images}:\;CNR=\frac{{\mathrm{SI}}_{tu\mathrm{mor}}-{SI}_{brain}}{\sqrt{\left({std\;{SI}_{tumor}}^{2}+{std\;{SI}_{brain}}^{2}\right)}}$$

where SI_tumor_ indicates tumor signal intensity in postinjection T1-weighted images (SIpost), SI_brain_ indicates healthy tissue SIpost, and std indicates standard deviation.

$$\mathrm{for}\;\mathrm{CEST}\;\mathrm{images}:\;CNR=\frac{{\mathrm{ST}}_{tumor}-{ST}_{brain}}{\sqrt{\left({std\;{ST}_{tumor}}^{2}+{std\;{ST}_{brain}}^{2}\right)}}$$

where ST_tumor_ indicates tumor CEST contrast in postinjection images (STpost), ST_brain_ indicates the healthy tissue CEST contrast in postinjection images (STpost), and std indicates standard deviation.

The lesion-to-brain ratio (LBR) between tumor and healthy brain tissue:

$$\mathrm{for}\;T1-\mathrm{weighted}\;\mathrm{images}:\;LBR=\frac{{SI}_{tumor}}{{SI}_{brain}}$$

$$\mathrm{for}\;\mathrm{CEST}\;\mathrm{images}:\;\mathrm{LBR}=\frac{{\Delta \mathrm{ST}}_{\mathrm{tumor}}}{{\Delta \mathrm{ST}}_{\mathrm{brain}}}$$

where ΔST_tumor_ indicates tumor CEST contrast in the difference contrast map (ΔST) and ΔST_brain_ indicates healthy tissue CEST contrast in the difference contrast map (ΔST).

The diagnostic capability to define tumor borders was assessed by comparing the tumor segmentations performed in T1-weighted postinjection images (Gdpost) with those in the CEST contrast postinjection images (STpost) and by comparing the segmented regions in contrast enhancement maps for Gadoteridol (Enh%) and in difference contrast map for Iopamidol (ΔST). Segmentation correspondence has been evaluated using similarity measures based on region overlap with the following metrics: Tanimoto coefficient (overlapping percentage), dice similarity coefficient (DICE), and volume similarity (volume ratio)^63^ (additional details and equations are provided in the Supporting Information).

Intrareader and inter-reader variabilities were assessed by analyzing CNR, overlapping percentage, and DICE on the whole set of images.

### Statistical Analysis

Data are expressed as mean ± standard deviation. GraphPad (Prism 9) was used for the statistical analyses. A *P*-value <0.05 was considered statistically significant. Intra- and interobserver agreements were assessed by calculating the intraclass correlation coefficients (ICCs). Agreement was considered excellent at ICC values *r* > 0.8, good at 0.6 < *r* ≤ 0.8, moderate at 0.4 < *r* ≤ 0.6, fair at 0.2 < *r* ≤ 0.4, and poor at *r* ≤ 0.2.

## Results

All the animals developed detectable tumors, with only one mouse that was not considered for analysis because the MRI scan was not exploitable for image quality. Therefore, a total of 9 mice were used for the analysis. Examples of preinjection and postinjection images following Gadoteridol injection (T1-weighted images pre- and postinjection and corresponding calculated contrast enhancement map—Enh%) or upon Iopamidol injection (and corresponding saturation transfer contrast map—ST%—before and after injection and difference CEST contrast map—ΔST) are shown in Figure 3. Images after Iopamidol or Gadoteridol injection provided enough contrast enhancement to enable clear tumor detection across the whole tumor volume.

**Figure 3:**
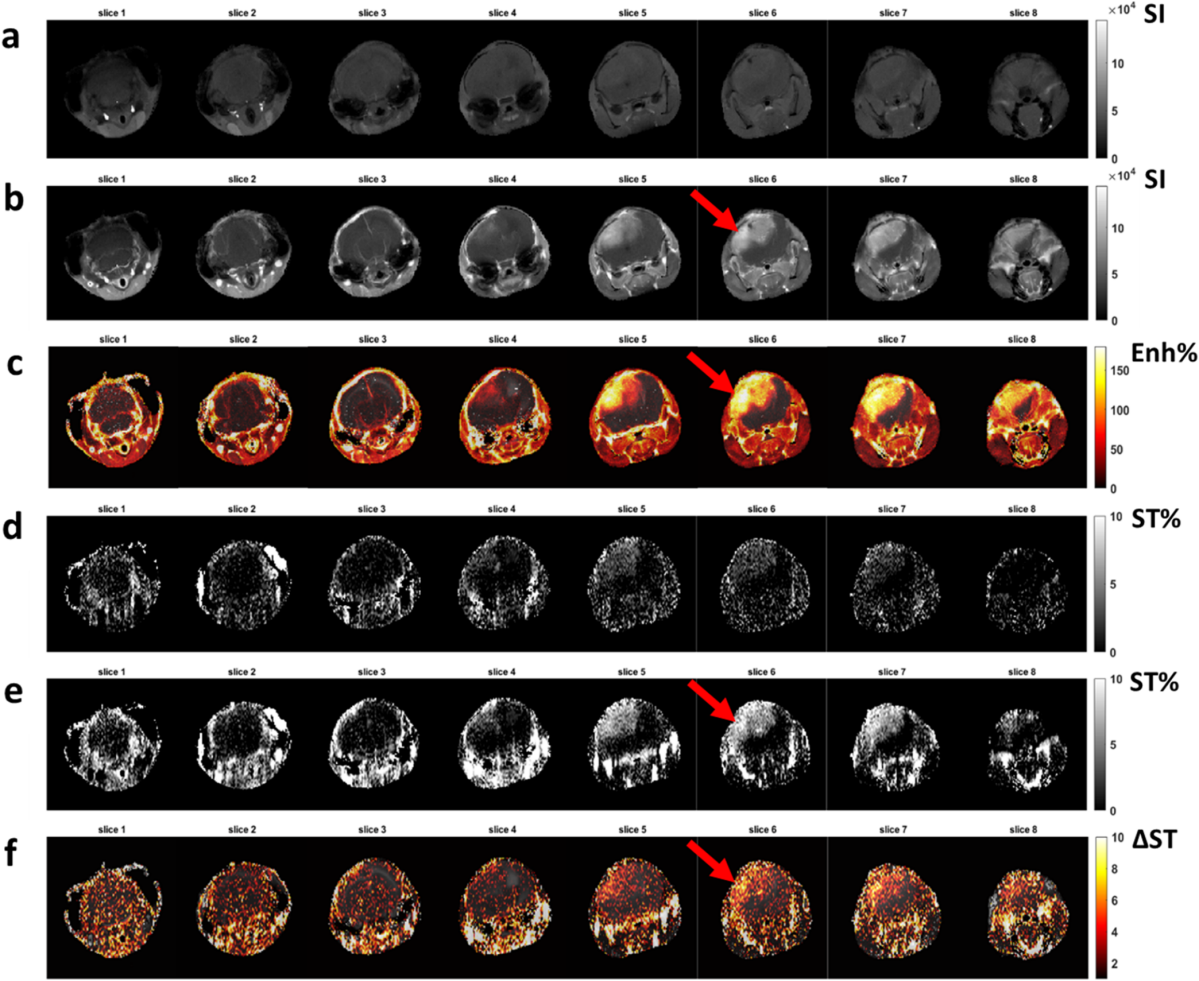
Representative (a) pre- and (b) postinjection images for Gadoteridol and (c) calculated percentage contrast enhancement (Enh%) map. Representative (d) pre- and (e) postinjection CEST contrast map (ST%) for Iopamidol and (f) calculated difference CEST (ΔST) contrast map. The arrows indicate lesions visible on the same slice after contrast agent administration.

### Qualitative Analysis

Examples of scoring by two experienced readers for tumor border delineation and tumor contrast enhancement are shown in Figure 4, for a representative patient with high score values (Figure 4a) and for a patient with low score values (Figure 4b). Overall results obtained by the two readers are summarized in Table 1. For Iopamidol-based images, tumor border delineation and contrast enhancements were rated above 3.6 or higher for postinjection CEST images (STpost), demonstrating good-to-excellent tumor border delineation and contrast enhancement, whereas difference CEST contrast maps (ΔST) showed lower diagnostic performance with scores slightly larger than 2. Gadoteridol obtained comparable scores to Iopamidol for postinjection T1-weighted images (Gdpost), whereas higher scores were obtained for contrast enhancement maps (Enh%) with excellent tumor delineation and high tumor enhancement with scores above 3.9. Qualitatively, diagnostic performance for Iopamidol was comparable to Gadoteridol for postinjection images.

**Figure 4:**
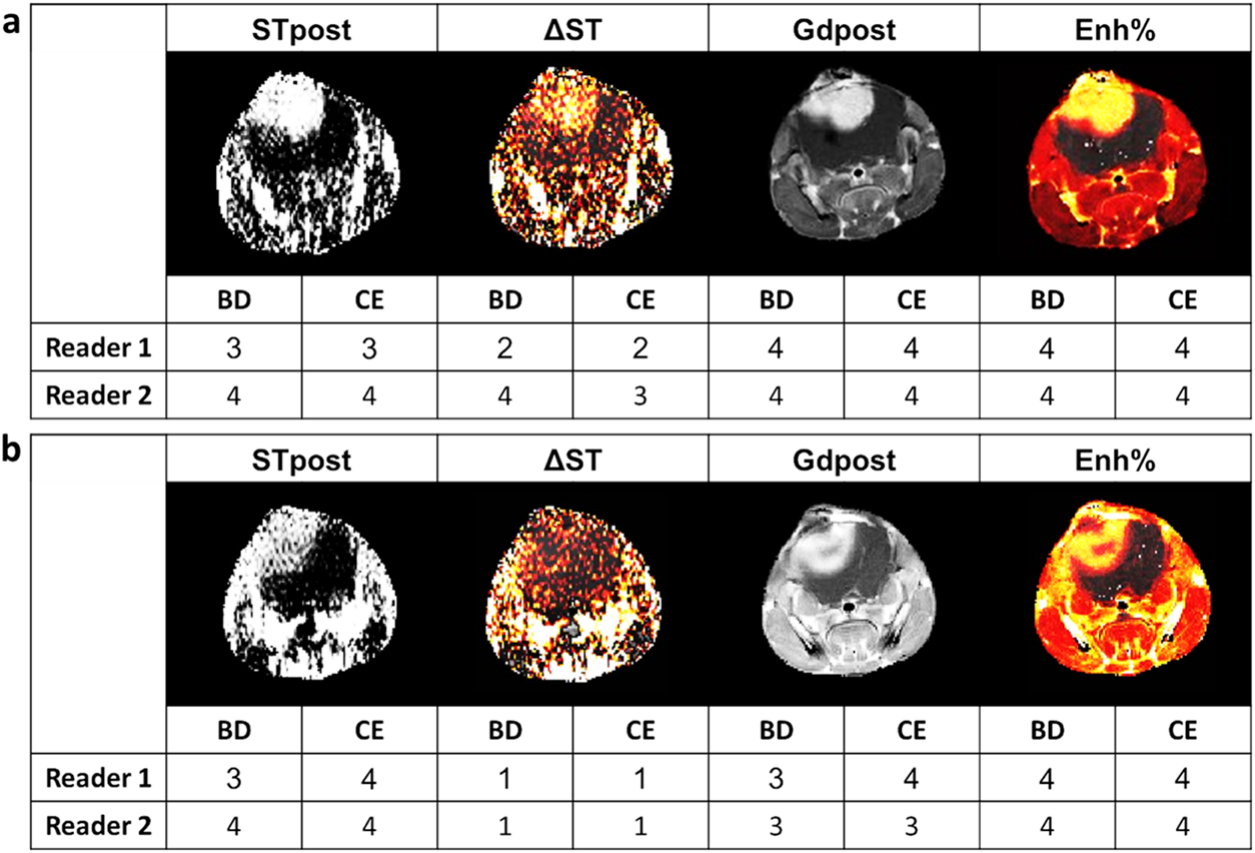
Visual assessment of postinjection contrast images (STpost and Gdpost for Iopamidol and Gadoteridol, respectively) and of calculated contrast map (ΔST and Enh% for Iopamidol and Gadoteridol, respectively) in patients with (a) high score or (b) low score. Images obtained after contrast agent injection are shown with examples of scores from two independent readers. BD = border delineation; CE = contrast enhancement (with the rating scale 1 = none or poor, 2 = moderate, 3 = good, 4 = excellent).

**Table 1:** Mean Scores of the Two Readers for the Qualitative Evaluation of Postcontrast Images (STpost and Gdpost) and of Calculated Contrast Maps (ΔST and Enh%) for Iopamidol and Gadoteridol, Respectively^a^

	STpost	ΔST	Gdpost	Enh%
border delineation	3.6 ± 0.5	2.2 ± 0.7	3.7 ± 0.5	3.9 ± 0.3
contrast enhancement	3.9 ± 0.2	2.1 ± 0.6	3.7 ± 0.5	3.3 ± 0.3

a Data are mean ± standard deviation. The rating scale has been described in the Methods section (briefly, a score of 1 = none or poor and a score of 4 = excellent).

Inter-reader variability was very small, with quotations of the two readers that were the same or differed for less than one point for 97% of the images for both tumor border delineation and contrast enhancement assessment for the two contrast agents.

### Quantitative Analysis—Contrast Enhancement

Determination of the quantitative enhancement after contrast agent injection was calculated for Iopamidol as difference CEST contrast map (ΔST) and for Gadoteridol as contrast enhancement (Enh%) and is presented in Figure 5. Although both molecules provided marked contrast enhancements inside the whole tumor region (2.3 ± 0.5% for Iopamidol and 100 ± 30% for Gadoteridol), the two values are not directly comparable because of the different equations exploited for the calculation. Therefore, two other quantitative metrics were used to assess the increase in contrast in the tumor region in comparison to that in the healthy brain region.

**Figure 5:**
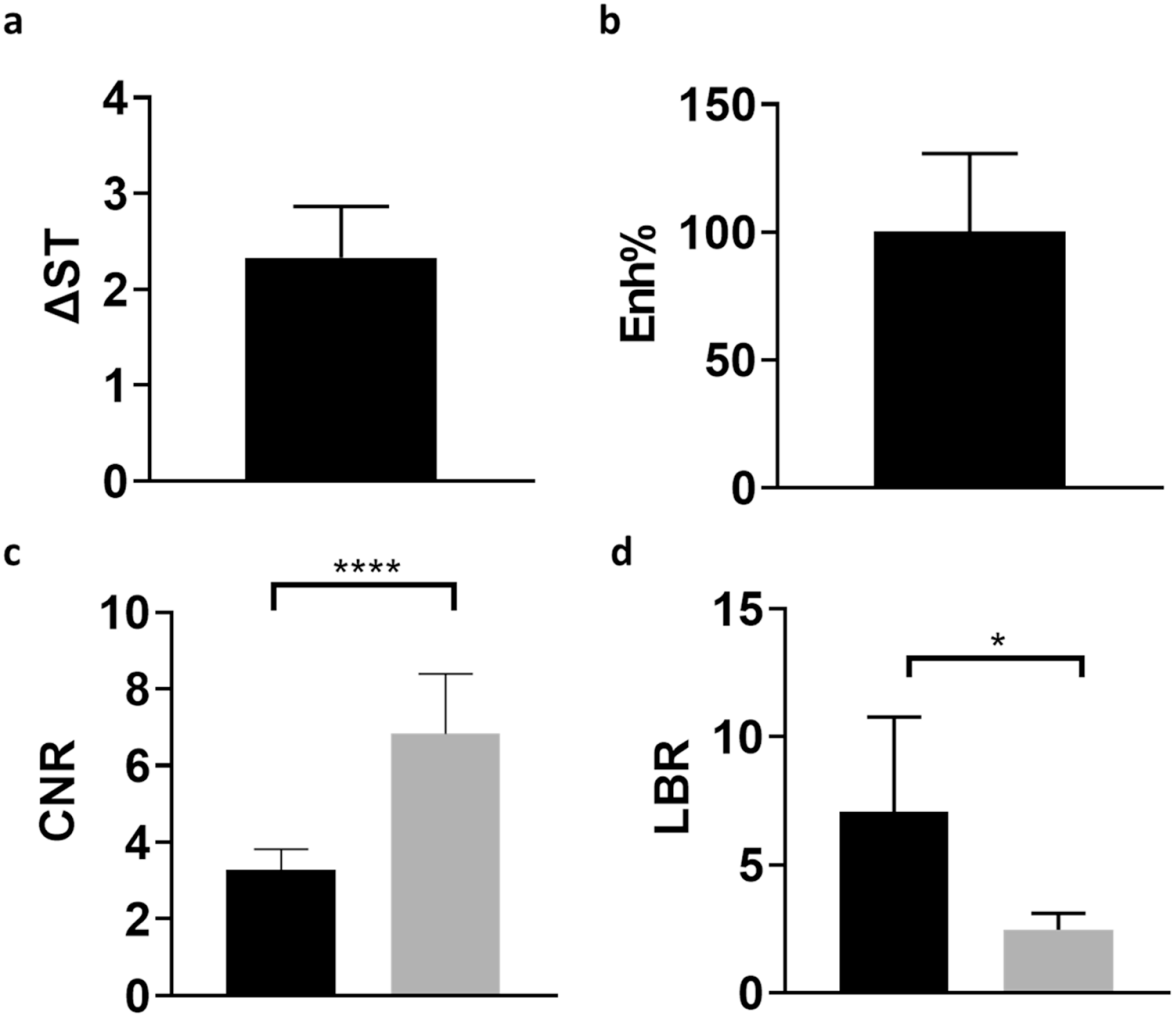
Quantitative determination of lesion enhancement comparison for Iopamidol (a) on difference contrast (ΔST) images versus Gadoteridol (b) on contrast-enhanced (Enh%) images, (c) for contrast-to-noise ratio (CNR) calculated on postinjection images for Iopamidol (STpost, black column) and Gadoteridol (Gdpost, gray column) and (d) for lesion-to-brain ratio (LBR) calculated for Iopamidol (black column) on difference contrast images (ΔST) and for Gadoteridol (gray column) on postinjection (Gdpost) images. * *p* < 0.05; **** *p* < 0.0001.

CNR values calculated between tumor and contralateral brain regions in postinjection images showed a marked increase for both Iopamidol and Gadoteridol (Figure 5c), confirming that both contrast agents can give enough contrast enhancement, highlighting the tumor area. CNR values after Gadoteridol injection were significantly 2.1-fold higher than after Iopamidol injection (CNR = 6.8 ± 1.6 vs 3.3 ± 1.0, *p* < 0.0001, respectively). On the other hand, LBR values calculated on difference contrast maps for Iopamidol (ΔST) and on postinjection images for Gadoteridol (Gdpost) showed an opposite trend with Iopamidol providing statistically significant 2.7-fold higher LBR values compared with Gadoteridol (6.2 ± 3.3 vs 2.3 ± 0.8, *p* = 0.015, respectively, Figure 5d). Although an opposite trend for CNR and LBR values was observed between Iopamidol and Gadoteridol, their absolute values are high enough for allowing a robust detection of the tumor region inside the brain.

### Quantitative Analysis—Tumor Border Delineation

Tumor lesions were delineated by drawing a region of interest in the enhancing region on both Gd-based and Iopamidol-based images (Figure 6). Tumor border delineation and tumor volume segmentation were evaluated on both postinjection images (STpost for Iopamidol and Gdpost for Gadoteridol, Figure 6a) and on contrast-enhanced images (ΔST for Iopamidol and Enh% for Gadoteridol, Figure 6b) by exploiting three different metrics to quantify the amount or the similarity of region overlapping. The mean calculated overlapping percentage values were 64 ± 6 and 72 ± 8% for postinjection images and contrast-enhanced maps, respectively (Figure 6c). The volume ratio metric was very high, with values of 0.94 ± 0.11 for both types of images (Figure 6d). High DICE similarity coefficient values were obtained for both postinjection images (0.83 ± 0.06) and for contrast-enhanced maps (0.78 ± 0.05, Figure 6e).

**Figure 6:**
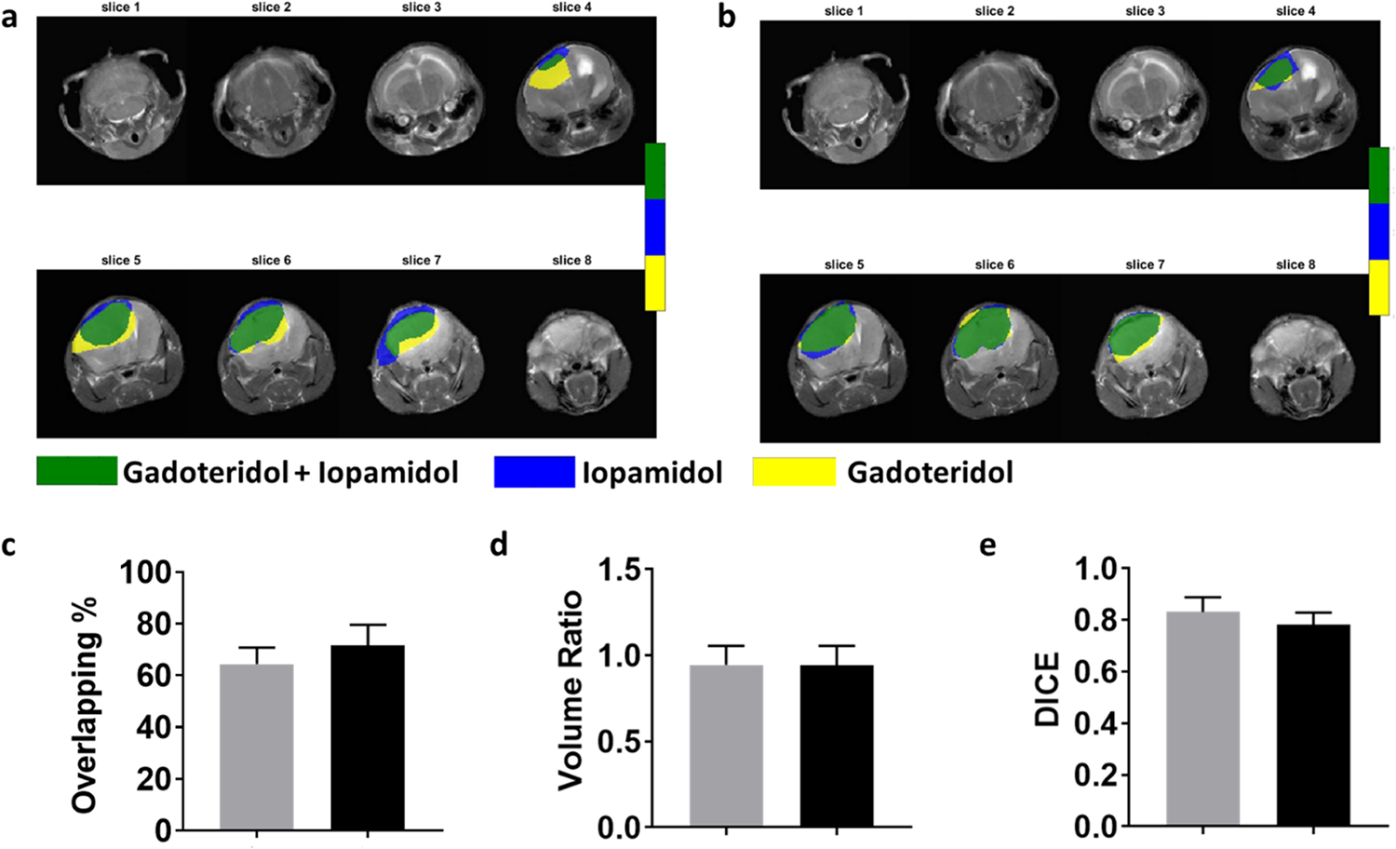
(a) Representative segmented regions delineating tumor lesions based on postinjection contrast images for Iopamidol (STpost, in blue) and for Gadoteridol (Gdpost, in yellow) and corresponding overlapping regions (in green) (b) and for segmented regions based on difference contrast maps for Iopamidol (ΔST, in blue) and for Gadoteridol (Enh%, in yellow) and corresponding overlapping regions (in green). Graphs showing the amount of overlapping of the segmented tumor regions between the postinjection images (in gray) for Gadoteridol (Gdpost) and Iopamidol (STpost) and between the contrast-enhanced maps (in black) for Gadoteridol (Enh%) and for Iopamidol (ΔST), in terms of (c) overlapping percentage (Tanimoto coefficient), (d) volume ratio, and (e) DICE similarity metrics.

Overall, when measured in terms of tumor border delineation, the performance with Iopamidol was similar to that achieved with Gadoteridol for all the calculated similarity metrics for both the postinjection and the contrast-enhanced images, suggesting comparable delineation of lesion borders.

### Intraobserver and Interobserver Variability

For intraobserver variability, we obtained an excellent agreement in the evaluation of CNR values (Figure 7a) with a intraclass correlation coefficient (ICC) of 0.96 (Table 2). A good correlation was obtained for the tumor border delineation metrics based on volume similarity with an ICC of 0.74 for both the overlapping percentage and DICE coefficient (Figures 7b,c and Table 2).

**Figure 7:**
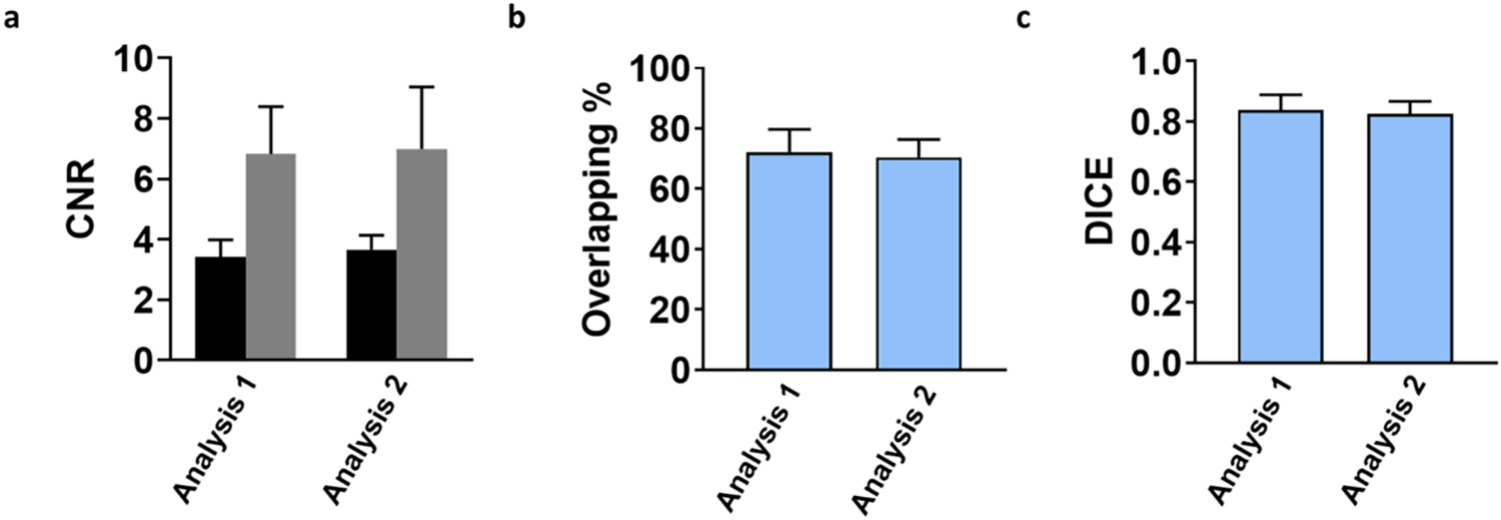
Quantitative parameters for (a) contrast-to-noise ratio (CNR) for Iopamidol (black column) versus Gadoteridol (gray column), (b) overlapping percentage, and (c) DICE similarity coefficient calculated for each patient by the same reader for the first (left) and second (right) analysis. Data are presented as bar graphs showing the mean ± SD (standard deviation).

**Table 2:** Intraobserver Analysis of Contrast-to-Noise Ratio (CNR) on Postinjection CEST (STpost) and T1-Weighted Images (Gdpost), and Overlapping Percentage and DICE Similarity Coefficient Metrics on Iopamidol Postinjection (STpost) Images and on Gadoteridol Postinjection (Gdpost) Images^a^

		analysis 1	analysis 2	ICC
CNR	***n***	18	18	**0.96**
	**mean**	5.1	5.3	
	**SD**	2.1	2.2	
overlapping percentage	***n***	9	9	**0.74**
	**mean**	72.2	70.5	
	**SD**	7.5	6	
DICE	***n***	9	9	**0.74**
	**mean**	0.84	0.82	
	**SD**	0.05	0.04	

a SD: standard deviation, ICC: intraclass correlation coefficient.

We observed similar results for the interobservers’ variability. A good to excellent inter-reader correlation was obtained in the evaluation of the CNR (Table 3) with an intraclass correlation coefficient of 0.7 (between readers 1 and 2), 0.99 (between readers 2 and 3), and 0.8 (between readers 1 and 3, Figure 8a). The tumor border delineation metrics showed fair to good correlation, with ICC values of 0.38 (between readers 1 and 2), 0.67 (between readers 2 and 3), and 0.40 (between readers 1 and 3) for the overlapping percentage metric (Figure 8b, Table 4) and with ICC of 0.34 (between readers 1 and 2), 0.63 (between readers 2 and 3), and 0.40 (between readers 1 and 3) for the DICE similarity coefficient (Figure 8c, Table 5).

**Figure 8:**
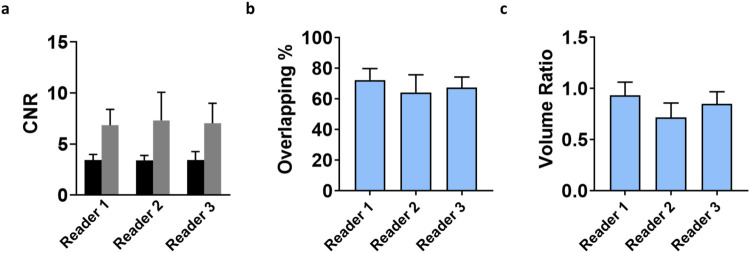
Quantitative parameters for (a) contrast-to-noise ratio (CNR) for Iopamidol (black column) versus Gadoteridol (gray column), (b) percentage overlapping, and (c) DICE similarity coefficient calculated for each patient by three independent readers. Data are presented as bar graphs showing mean ± SD (standard deviation).

**Table 3:** Interobservers’ Analysis of Contrast-to-Noise Ratio (CNR) on Postinjection Images for Iopamidol (STpost) and for Gadoteridol T1-Weighted (Gdpost) Images^a^

CNR		reader 1	reader 2	reader 3	ICC
reader 1 vs reader 2	***n***	18	18		**0.70**
	**mean**	5.0	5.3		
	**SD**	2.1	2.8		
reader 2 vs reader 3	***n***		18	18	**0.99**
	**mean**		5.3	5.2	
	**SD**		2.8	2.3	
reader 1 vs reader 3	***n***	18		18	**0.80**
	**mean**	5.0		5.2	
	**SD**	2.1		2.3	

a SD: standard deviation, ICC: intraclass correlation coefficient.

**Table 4:** Interobservers’ Analysis of the Overlapping Percentage Metric on Iopamidol Postinjection (STpost) Images and on Gadoteridol Postinjection (Gdpost) Images^a^

overlapping percentage		reader 1	reader 2	reader 3	ICC
reader 1 vs reader 2	***n***	9	9		**0.38**
	**mean**	72	64		
	**SD**	7	11		
reader 2 vs reader 3	***n***		9	9	**0.67**
	**mean**		64	67	
	**SD**		11	7	
reader 1 vs reader 3	***n***	9		9	**0.40**
	**mean**	72		67	
	**SD**	7		7	

a SD: standard deviation, ICC: intraclass correlation coefficient.

**Table 5:** Interobservers’ Analysis of the DICE on Iopamidol Postinjection (STpost) Images and on Gadoteridol Postinjection (Gdpost) Images^a^

DICE		reader 1	reader 2	reader 3	ICC
reader 1 vs reader 2	***n***	9	9		**0.34**
	**mean**	0.84	0.78		
	**SD**	0.05	0.09		
reader 2 vs reader 3	***n***		9	9	**0.63**
	**mean**		0.78	0.80	
	**SD**		0.09	0.05	
reader 1 vs reader 3	***n***	9		9	**0.40**
	**mean**	0.84		0.80	
	**SD**	0.05		0.05	

a SD: standard deviation, ICC: intraclass correlation coefficient.

## Discussion

Contrast-enhanced MRI provides essential information to detect and characterize brain tumors, by increasing the contrast difference between normal and abnormal tissues, allowing the selection of the best treatment strategy for patients.^64^ However, concerns related to the gadolinium accumulation in the brain upon multiple administration, although without clinically consequences, brought to the development of novel approaches, including more efficient (i.e., with higher relaxivity) GBCAs, artificial intelligence methods, or new contrast mechanism exploiting gadolinium-free contrast agents.^65^

Since contrast-enhancement capabilities are dependent on the abnormal vasculature or on impaired blood–brain barrier regions, administered iodinated contrast media can provide similar information to GBCAs, although with limited soft tissue contrast within the CT modality.^66,67^ We hypothesize that the combination of an iodinated contrast medium, Iopamidol, with the MRI-CEST technique may provide similar diagnostic information to current GBCA for detecting brain tumor lesions with improved tissue contrast.

The current study used a glioblastoma murine model to compare the diagnostic performance of Iopamidol for detecting primary tumor lesions and for delineating the tumor border with that of Gadoteridol, as a reference extracellular GBCA. We demonstrated that Iopamidol can provide lesion detection and visualization similar to that of Gadoteridol, although with lower contrast enhancement capabilities.

Qualitative analysis showed that overall tumor border delineation was higher with Gadoteridol (qualitative score >3.7 for both postinjection and contrast-enhanced images) as compared with Iopamidol (qualitative score >3.5 only for the postinjection image). In addition, overall tumor contrast enhancement was slightly higher with Iopamidol only in postcontrast image (qualitative score of 3.9) as compared with Gadoteridol (qualitative score of 3.7). Although contrast enhancement values are not directly comparable between the two contrast agents because of the differences in their calculation (ΔST for Iopamidol and Enh% for Gadoteridol), quantitative analyses exploiting the CNR and LBR metrics provided additional useful findings. In particular, CNR values were 2-fold higher with Gadoteridol than Iopamidol, whereas LBR values were more than 2-fold higher for Iopamidol than for Gadoteridol, thus explaining the same differences observed in the qualitative results. The LBR metric is based on the ratio of the contrast between the tumor and the healthy regions (and not on the difference as for the CNR metric), therefore providing a more robust estimation of the diagnostic performance of the two contrast agents. Interestingly, a CNR higher than 2, as observed for Iopamidol, is conventionally considered reliable for detecting tumor lesions.^68^

A precise delineation of border lesions is beneficial for follow-up studies and patient management. In this study, tumor border delineation and tumor volume segmentation were compared between Iopamidol and Gadoteridol on both postinjection images and on contrast-enhanced maps. To allow a more reliable comparison of the segmented volumes, the two contrast agents were administered during the same MRI session, with a 30 min interval, considered sufficient for the first contrast agent washout.^57^ Furthermore, this approach allows a pixel-by-pixel comparison between the two segmented images obtained upon Iopamidol or Gadoteridol injection and avoids any tumor volume change due to tumor growth. The average DICE metric and the volume ratio metrics showed high similarities between the two manually drawn tumor regions for both Iopamidol- and Gadoteridol-derived segmented regions, hence suggesting high accuracy in tumor border delineation based on Iopamidol. Similar results have been observed between Iopamidol and Gadoteridol, although in a subcutaneous breast murine model.^57^ Moreover, although the qualitative analysis showed a slightly reduced diagnostic performance when exploiting the ΔST maps in contrast to the postinjection ST maps (Table 1), that could be explained by an increased dependence of the subtraction technique with small movement artifacts on a pixel-by-pixel basis, overall the quantitative comparison of the similarities between the tumor segmented regions did not show any significant difference (Figure 6c–e). Of note, the exploitation of only the postinjection ST images might represent a new and more viable approach for the clinical implementation of this CEST contrast agent, avoiding the acquisition of two CEST scans, hence reducing both acquisition time and movement artifacts.

This study has been performed with a high-field 7T scanner that increases the CEST sensitivity to Iopamidol; however, several studies have already demonstrated the clinical translatability of Iopamidol (or of similar iodinated contrast media) at the lower magnetic field strength of 3T.^69,70^ Of note, iodinated contrast media within the MRI-CEST imaging modality have proven tumor detection capability on clinical MRI scanners with comparable CEST contrast enhancement as observed in this study.^52,71^ Moreover, the moderate contrast efficiency of Iopamidol could be further increased by investigating optimized saturation pulse shapes or by novel deep-learning approaches tailored for CEST images.^72–77^

Compared to Iopamidol, a low-molecular-weight (1 kDa) dextran showed good contrast capability to detect tumor lesion in the same GL261 glioblastoma murine model, with similar findings when compared to a gadolinium-based contrast agent, although showing lower CEST contrast (ΔST ≈ 1%) in the tumor region.^36^ Of note, molecular size and charge properties can largely affect the extravasation and the contrast capabilities, as shown by investigating dextran molecules with different molecular weights.^38^ To overcome these issues, and for providing a robust and fair comparison of the contrast properties of Iopamidol, we selected Gadoteridol as representative of GBCA because of (i) both molecules are nonionic, (ii) they have a similar molecular weight (777 Da for Iopamidol and 558.7 Da for Gadoteridol), (iii) the pharmacokinetic is similar (elimination half-life of 2 h for Iopamidol and of 1.57 h for Gadoteridol), (iv) they are completed excreted by renal filtration (90 and 94% of the injected dose in urine for Iopamidol and for Gadoteridol, respectively, 24 h after intravenous administration) and (v) both molecules did not undergo metabolization.^78,79^

Interobserver results showed a similar range of 0.29 for the ICC values of all the metrics (Tables 3–5), although the overlapping percentage and the DICE metrics showed lower absolute ICC values than the CNR metric, likely because the latter is less dependent on tumor border delineation.

Clinical translation of iodinated contrast media from CT to MRI should also take into account the differences in dose, cost-effectiveness, and safety profiles when compared to conventional GBCAs. In particular, administered doses for iodinated contrast media are usually larger than for GBCAs, when considering the amount of injected material (ca. 4 g of GBCA vs 35 g of iodinated contrast media), although the cost for dose is lower for iodinated contrast media. Moreover, the safety profiles of both classes are considered very high, regardless of the type of the compound, although iodinated contrast media have lower nephrotoxicity than GBCAs for an equivalent dose.^80,81^

This study has several limitations. The overall acquisition time for the CEST images is 5 times larger than that required for T1-weighted images at the same volume coverage and spatial resolution that could limit clinical translatability. However, at the clinical level, fast CEST sequences with 3D full brain coverage are already available with shorter acquisition times.^82–85^ Moreover, long acquisition time could potentially increase movement artifacts that have not been corrected for in this study, but motion correction techniques for CEST images could be potentially exploited in further studies.^86,87^ Furthermore, histological validation of tumor borders was not performed because the sequential administration of the two contrast agents within the same mouse hampered a matched comparison with the acquired MR images.

Additionally, on clinical scanner operating at lower magnetic field strengths, the CEST contrast efficiency of Iopamidol is expected to be lower than at higher field strengths, although several studies demonstrated good CEST contrast detection, hence making this approach potentially feasible at 3T.^52,69^

## Conclusions

In conclusion, Iopamidol showed comparable capability to GBCAs to detect brain tumors and similar diagnostic precision to delineate tumor borders, although with lower contrast efficiency. Further improvements in CEST contrast quantification and additional studies are still needed to assess the full potential of Iopamidol for brain tumor imaging with the CEST MRI technique.

## Supplementary Material



## Notes

Description of the segmentation similarity metrics (PDF)

## Abbreviations

GBCA: gadolinium-based contrast agent
CEST: chemical exchange saturation transfer
CNR: contrast-to-noise ratio
LBR: lesion brain ratio
ST: saturation transfer
SI: signal intensity
