# Supplementary material for: Brain Tumor Imaging with Iopamidol CEST MRI: In Vivo Detection and Validation
Source: Chem Biomed Imaging. 2025 Sep 23;4(3):354–65. doi: 10.1021/cbmi.5c00101 (PMC13014332; doi:10.1021/cbmi.5c00101)
Supplement: Supplementary file 1 [file im5c00101_si_001.pdf]

Supporting Information

Brain tumor imaging with Iopamidol CEST MRI: in vivo detection and validation

Elena Botto, MSc,[1] Antonella Carella, PhD,[1] Francesco Gammaraccio, MSc,[2] Daisy Villano, MSc,[2] Riccardo Gambino, MSc,[2] Alessia Corrado, MSc,[1] Elisa Pirotta, MSc,[1] Feriel Romdhane, PhD,[1] and Dario Livio Longo, PhD,[1*]

[1] Institute of Biostructures and Bioimaging (IBB), National Research Council of Italy (CNR), Via Nizza 52, 10126, Torino, Italy

[2] Department of Molecular Biotechnology and Health Sciences, University of Turin, Via Nizza 52, 10126 Turin, Italy

*Correspondence to: Dario Livio Longo, Istituto di Biostrutture e Bioimmagini (IBB) – Consiglio Nazionale delle Ricerche (CNR), Via Nizza 52, 10126, Torino, Italy. E-mail: dariolivio.longo@cnr.it

Segmentation similarity metrics

Segmentation has been evaluated using similarity measures based on region overlap of the two manually-drawn regions of interest in the CEST (region A) and T1-weighted images (region B).

The Tanimoto coefficient (Overlapping Percentage) is defined as the ratio of the number of voxels in the intersection of A and B to the number of voxels in their union (1). This can be represented by the percentage formula:

$$Overlapping\ Percentage = \left(\frac{N(A \cap B)}{N(A \cup B)}\right) \times 100$$

where *A* and *B* are the two overlapping regions, *N()* is the number of voxels in the enclosed set.

The Dice Similarity Coefficient (DICE) is defined as the ratio of the number in the intersection to the mean label volume (1):

$$DICE = \frac{2N(A \cap B)}{N(A) + N(B)}$$

where A and B are the two overlapping regions, N() is the number of voxels in the enclosed set.

The Volume Ratio is defined as the ratio between the number of voxels of region A and the number of voxels of region B:

$$Volume\ Ratio = \frac{V_A}{V_B}$$

where V_A is the number of voxels of region A and V_B is the number of voxels of region B.

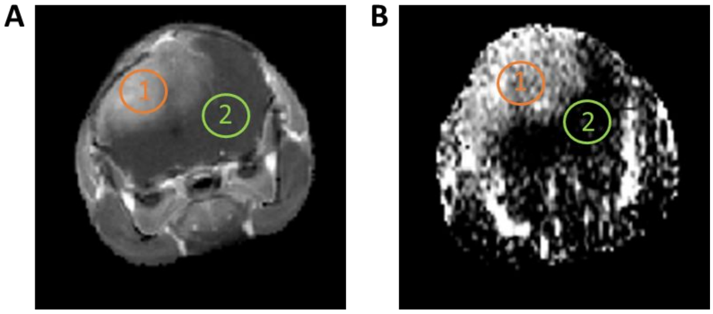

Figure S1. Example of region of interest (ROI) drawn in the Gadoteridol post-injection image (Gdpost, A) and on the Iopamidol post-injection image (STpost, B) for calculating quantitative contrast enhancment metrics (CNR and LBR). ROI number 1 represents tumor area and ROI number 2 represents healthy tissue.

References

1. Crum WR, Camara O, Hill DL**.** Generalized overlap measures for evaluation and validation in medical image analysis. IEEE Trans Med Imaging. 2006;25(11):1451-61.
